# A bioinformatic analysis of ribonucleotide reductase genes in phage genomes and metagenomes

**DOI:** 10.1186/1471-2148-13-33

**Published:** 2013-02-07

**Authors:** Bhakti Dwivedi, Bingjie Xue, Daniel Lundin, Robert A Edwards, Mya Breitbart

**Affiliations:** 1 College of Marine Science, University of South Florida, St. Petersburg, FL 33701, USA; 2 School of Biotechnology, KTH Royal Institute of Technology, Stockholm, Sweden; 3 Department of Computer Sciences, San Diego State University, San Diego, CA, 92182, USA

**Keywords:** Ribonucleotide reductase, Phage, Metagenome, Phage metadata, Phylogenetics, Evolution, Split gene

## Abstract

**Background:**

Ribonucleotide reductase (RNR), the enzyme responsible for the formation of deoxyribonucleotides from ribonucleotides, is found in all domains of life and many viral genomes. RNRs are also amongst the most abundant genes identified in environmental metagenomes. This study focused on understanding the distribution, diversity, and evolution of RNRs in phages (viruses that infect bacteria). Hidden Markov Model profiles were used to analyze the proteins encoded by 685 completely sequenced double-stranded DNA phages and 22 environmental viral metagenomes to identify RNR homologs in cultured phages and uncultured viral communities, respectively.

**Results:**

RNRs were identified in 128 phage genomes, nearly tripling the number of phages known to encode RNRs. Class I RNR was the most common RNR class observed in phages (70%), followed by class II (29%) and class III (28%). Twenty-eight percent of the phages contained genes belonging to multiple RNR classes. RNR class distribution varied according to phage type, isolation environment, and the host’s ability to utilize oxygen. The majority of the phages containing RNRs are *Myoviridae* (65%), followed by *Siphoviridae* (30%) and *Podoviridae* (3%). The phylogeny and genomic organization of phage and host RNRs reveal several distinct evolutionary scenarios involving horizontal gene transfer, co-evolution, and differential selection pressure. Several putative split RNR genes interrupted by self-splicing introns or inteins were identified, providing further evidence for the role of frequent genetic exchange. Finally, viral metagenomic data indicate that RNRs are prevalent and highly dynamic in uncultured viral communities, necessitating future research to determine the environmental conditions under which RNRs provide a selective advantage.

**Conclusions:**

This comprehensive study describes the distribution, diversity, and evolution of RNRs in phage genomes and environmental viral metagenomes. The distinct distributions of specific RNR classes amongst phages, combined with the various evolutionary scenarios predicted from RNR phylogenies suggest multiple inheritance sources and different selective forces for RNRs in phages. This study significantly improves our understanding of phage RNRs, providing insight into the diversity and evolution of this important auxiliary metabolic gene as well as the evolution of phages in response to their bacterial hosts and environments.

## Background

Phages (viruses that infect bacteria) are the most abundant biological entities on Earth, and the vast majority of known phages contain double-stranded DNA (dsDNA) genomes [1-3]. As the number of fully sequenced phage genomes and environmental metagenomes increases, our knowledge about phages at molecular, evolutionary, and ecological levels improves significantly. Over the past decade, phage genomes have been shown to contain host-like metabolic genes known as auxiliary metabolic genes (AMGs) that encode proteins involved in photosynthesis, carbon metabolism, phosphate acquisition, and nucleotide metabolism [4-16]. Although the impact of many of these AMGs on phage success has not been experimentally demonstrated, it is thought that expression of these genes supports host metabolism throughout the infection process. For example, studies have shown expression of cyanophage-encoded photosynthesis (*psbA*), carbon metabolism (*talC, zwf, gnd, cp12*), and nucleotide metabolism (*nrdA, nrdB, nrdJ*) genes during infection, thereby sustaining host metabolism and promoting phage replication [17,18]. In contrast to core phage genes (i.e., those found only in phage genomes, such as structural genes), phages presumably acquire AMGs from their hosts as an adaptation to certain environmental conditions, making AMGs perfect candidate genes for studying the evolution of phages in response to specific hosts and environments.

One of the most common AMGs is the ribonucleotide reductase (RNR) gene involved in nucleotide biosynthesis [9,12,16]. RNRs are the enzymes responsible for converting ribonucleotides to deoxyribonucleotides and are therefore critical for DNA replication and repair in all domains of life [19-21]. Three main classes of RNRs, denoted as class I, II, and III, are known [19,21]. Class I RNR is oxygen dependent and only found in organisms that can grow aerobically. Class I RNR is commonly divided into two subclasses, Ia and Ib, which are encoded by two (*nrdA* and *nrdB*) and four (*nrdH, nrdI nrdE,* and *nrdF*) different genes, respectively. Some studies suggest that this sub-division is not an accurate representation of the subclasses within class I RNR [22,23] and therefore needs to be re-evaluated. However, given the lack of consensus, the commonly accepted subclasses (class Ia and class Ib) were used in this study. Class Ia and Ib RNRs are distantly related to each other and share sequence motifs. Class II RNR is encoded by a single gene, *nrdJ*. As this RNR class is oxygen independent, it is often found in facultative anaerobes and strict anaerobic organisms. The *nrdJ* gene is distantly related to the *nrdA* and *nrdE* genes of class I RNRs. Class III RNR is encoded by the *nrdD* and *nrdG* genes. This RNR class is oxygen intolerant and therefore is present in organisms that grow anaerobically. The *nrdDG* genes do not share sequence similarity to either class I or class II RNR genes.

The presence of RNRs has been reported in a wide range of cultured phage genomes, including phages infecting both autotrophic and heterotrophic bacterial hosts, and members of all families of the tailed dsDNA *Caudovirales* (i.e., *Podoviridae*, *Myoviridae*, *Siphoviridae*) [4,8,11-13,16,24-27]. In addition, RNRs are amongst the most abundant genes identified in marine viral metagenomes [28,29]. Establishment of the manually curated RNR database (RNRdb; http://rnrdb.molbio.su.se/) enabled careful investigation of the diversity of RNR classes in all cellular organisms as well as viruses [20]. Since the creation of the RNRdb, the number of dsDNA phages with sequenced genomes has grown considerably, increasing to 685 as of July 2011 (http://www.phantome.org/).

This study determined the presence of different RNR classes in all available dsDNA phage genomes and compared RNR distribution to metadata including the phage family, original environment of isolation, bacterial host specificity, and oxygen requirements of known hosts. Phylogenetic trees were constructed to investigate the evolutionary relationships between RNRs from phages and their bacterial hosts, since horizontal gene transfer has been demonstrated to play an important role in RNR evolution [20,24,30]. Several putative split RNR genes interrupted by intervening sequences of self-splicing introns or inteins were identified, providing further evidence for the role of frequent genetic exchange. Finally, the distribution of RNRs in environmental viral metagenomes was examined and compared to the types of RNRs found in cultured phage genomes. This comprehensive study significantly improves our understanding of RNRs in phages, providing insight into the diversity and evolution of this important auxiliary metabolic gene.

## Results and discussion

### RNRs in completely sequenced dsDNA phage genomes

#### Distribution of RNR classes

**Figure 1:**
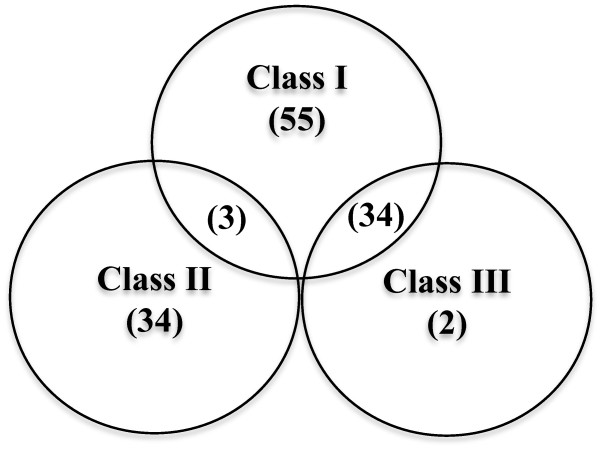
**An overview of the RNR classes detected in completely sequenced phage genomes.** The number within parenthesis represents the number of phages containing one or more RNR genes belonging to that particular class or class combination, for a total of 128 phages with detectable RNR genes.

Of the 685 fully sequenced dsDNA phage genomes examined in this study, 128 phages (18.7%) contained identifiable RNR genes. Figure 1 shows the overall distribution of RNR classes in these phages, where a phage is considered positive for a given class if it contains one or more RNR genes belonging to that class. The most commonly observed RNR genes belonged to class I (found in 70% of the sequenced phage genomes containing RNR genes), followed by class II (29%), then class III (28%). Forty-three percent of the phage genomes contained only class I RNRs, while 27% contained only class II, and 1% contained only class III genes. Twenty-eight percent of the phage genomes containing RNRs had a combination of RNR classes, with 27% containing genes belonging to both class I and III RNRs and 2% containing genes belonging to both class I and II. No phage genomes containing both class II and III genes were identified. The pattern of RNR classes observed in this study is consistent with the phage RNR distribution in the RNRdb [20]; however, the current study nearly tripled the number of phages known to contain RNR genes and identified phages containing both class I and II RNR genes, a combination that has not been previously reported in phages. These findings indicate that as more phages are sequenced and annotated in the future, our perception of RNR diversity, distribution, and evolution in phages could change significantly.

Compared to their bacterial hosts [20], a higher proportion of phages contain only class I RNR (43% of phages with RNRs but only 29% of bacteria) or only class II RNR (27% of phages with RNRs but only 12% of bacteria). While approximately 14% of bacteria contain either genes from all three RNR classes or both class II and III RNR genes, these combinations have not yet been observed in phage genomes. In addition, a much smaller proportion of phage genomes contain both class I and II genes (2% of phages with RNRs) compared to their bacterial hosts (11% of bacteria). There are several potential explanations for the differential distribution of RNR classes in phages versus bacteria. The smaller genome size of phages may apply selective pressure on the acquisition and maintenance of AMGs; therefore, it is likely that RNR genes are only maintained if they provide a significant advantage to phage replication. In addition, it is possible that phages are more likely to carry genes encoding rate-limiting steps, those that encode rapidly-degrading proteins, or genes complementing the suite of genes found in their hosts. Additionally, certain RNR genes may be more likely to be transferred due to their genomic context or the presence of sequences such as homing endonucleases (see below). However, it is also important to note that the results may be affected by database biases resulting from the hosts or environments from which the sequenced phages have been isolated. Future studies need to examine patterns of gene expression of phage RNRs throughout the infection cycle under different environmental conditions in order to elucidate the conditions under which encoding RNRs provides an advantage to phages.

#### RNR distribution compared to phage family, isolation source, and oxygen requirements of known hosts

**Figure 2:**
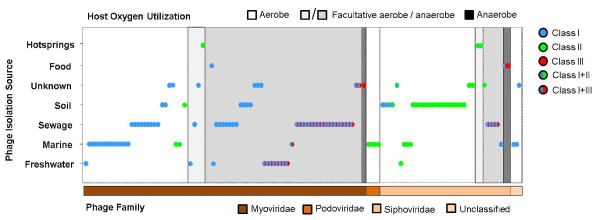
**Distribution of RNR classes in completely sequenced phage genomes displayed by phage family, environmental source of isolation, and host oxygen requirements.** Each circle represents a phage categorized by phage family on the bottom horizontal axis, environmental isolation source on the vertical axis, and the oxygen consumption ability of its known bacterial host on the top horizontal axis. Each circle represents a single phage and is color-coded to reflect the RNR class or class combination identified in that particular phage.

The distribution of different RNR classes in phages was compared to available information about the phage family, environmental isolation source, and oxygen requirements of known bacterial hosts in order to determine the effects of each of these factors (Figure 2). The majority (65%) of the phages containing RNR genes belong to the *Myoviridae*, which only comprise 23% of the 685 completely sequenced phage genomes. Despite the fact that *Siphoviridae* is the most common phage family in the database (comprising 57% of the available genomes), only 30% of the phages containing RNRs belong to the *Siphoviridae*. Only 3% of the genomes containing RNR genes are *Podoviridae* (despite the fact that this family accounts for 15% of the completely sequenced phage genomes), and the remaining 2% of the RNR-containing phages are unclassified (Additional file 1: Table S1). The RNR distribution amongst completely sequenced phage genomes may be driven by genome size, as *Myoviridae* generally contain larger genomes (average = 184 kb) than *Siphoviridae* and *Podoviridae* (average = 52 kb and 42 kb, respectively). However, it is important to remember that the database is extremely biased towards particular host types and environments (e.g., approximately 40% of the *Siphoviridae* were isolated from soil on *Mycobacterium smegmatis*). It is possible that phages infecting certain hosts or isolated from certain environments may be more or less likely to contain RNR genes depending on host metabolism or environmental selective pressures since RNRs likely provide evolutionary advantages for phage success under certain conditions (e.g., where phosphorus is limited and nucleotide recycling is critical) [18]. It would also be interesting to investigate how RNR distribution is affected by the nature of the phage life cycle (i.e., lytic versus temperate), although the linkage between lifestyle and phage family will make this feature difficult to discriminate (e.g., *Siphoviridae* are frequently temperate, while the majority of *Podoviridae* are lytic).

Phage family appears to have a strong effect on the class of RNR present. The majority of RNR genes in *Myoviridae* belong to class I, and class I+III, with very few *Myoviridae* containing class II RNR genes (Figure 2). In contrast, class II RNR was the only class identified amongst the four *Podoviridae* phages with RNR genes. All members of the *Podoviridae* containing RNR genes were isolated from the marine environment and infect *Roseobacter* and *Synechococcus* (see Additional file 1: Table S1; [4,11,12,27,31]). The co-existing hosts for these *Podoviridae* also possess the class II *nrdJ* gene, creating the possibility that by encoding *nrdJ*, these phages gain a competitive advantage in the nutrient-limited marine environment. The fact that *Podoviridae* exclusively contain class II RNR may also be driven by the fact that this RNR class only requires a single gene (*nrdJ*), which may be a selective advantage for the relatively small *Podoviridae* genomes. However, *Siphoviridae* are also primarily dominated by class II *nrdJ*, particularly amongst phages that infect aerobic hosts (Figure 2).

To determine the effect of isolation environment, the sources from which the phages were initially isolated were determined from the literature and categorized as marine, freshwater, sewage, hot springs, soil, food, or unknown. No single environmental source exclusively contained phages with only one RNR class; however, several trends were observed in the data. Phages from the marine environment primarily contain either class I or class II RNR (Figure 2). The only exception to this trend was *Vibrio parahaemolyticus* phage KVP40, a broad host range phage that was isolated from polluted seawater [32], which contains genes for both class I and III RNRs. This is reminiscent of phages from sewage, which are dominated by class I RNR or class I+III RNR, with no class II RNR genes detected. In contrast, phages from soil are dominated by class II RNR, with several instances of class I or I+II, but no class III RNR genes. Although only a few genomes are available, all phages isolated from hot springs with RNRs contain class II RNR.

The oxygen requirements of the isolation host for each phage also appear to influence the RNR class present, consistent with the observation that class I RNR is aerobic, class II is oxygen independent, and class III is anaerobic [19,21]. Regardless of isolation environment or phage family, phages infecting aerobes contain primarily class I or class II RNR genes (Figure 2). Most of the phages that infect facultative anaerobes contain class I+III genes, and all three phages that contain only class III RNR genes are known to infect strict anaerobes (*Clostridium* spp.). In bacteria, the class II *nrdJ* gene can enable DNA replication and repair under oxygen-limiting conditions, potentially explaining the presence of class II RNR in some phages infecting facultative aerobes and facultative anaerobes. The class II+III combination was completely absent amongst the 685 phage genomes analyzed, suggesting that the combination of class I+II or class I+III is more advantageous for infection under fluctuating oxygen conditions. However, it is important to note that very few genomes of phages infecting anaerobes are available, making it possible that the class II+III combination may be discovered in phages as more genomes are sequenced.

The class I+II RNR combination was fairly rare, and observed in phages for the first time in this study. Three *Mycobacterium* phages (Che12, D29, and L5) isolated from soil contain this RNR combination. All three of these phages infect the same host, *Mycobacterium tuberculosis*, which carries a similar suite of genes. Surprisingly, these are the only *Mycobacterium* phages with strong hits to the *nrdH* gene in addition to the *nrdJ* gene encoded by other *Siphoviridae* that infect *Mycobacterium* spp. (Additional file 1: Table S1 and Figure 2). Hence, it seems likely that *Mycobacterium* phages acquired *nrdJ* from their bacterial counterparts a long time ago, which then evolved independently in the respective phage lineages (see phylogenetic analysis below). Additional lateral gene transfers then likely occurred in phages infecting *M. tuberculosis*, resulting in the acquisition of a subset of class Ib genes from their host. Future studies need to determine whether the presence of only a few class Ib genes rather than the entire set (*nrdHIEF*) sufficiently confers a selective advantage with respect to phage replication. Two of the phages (Myrna and Pumpkin) infecting *M. smegmatis* contain class Ib genes (*nrdHF* and *nrdH*, respectively), but lack the class II *nrdJ* gene (Additional file 1: Table S1). It should be noted that the majority of the *Mycobacterium* phage genomes analyzed in the study showed HMMER similarities to *nrdH* as the only RNR gene, but these were considered false positives due to the high similarity between *nrdH* and thioredoxins/glutaredoxins [33,34] and were therefore removed from the analysis. More work is needed to confirm the functional identity of these genes.

#### Gene organization

**Figure 3:**
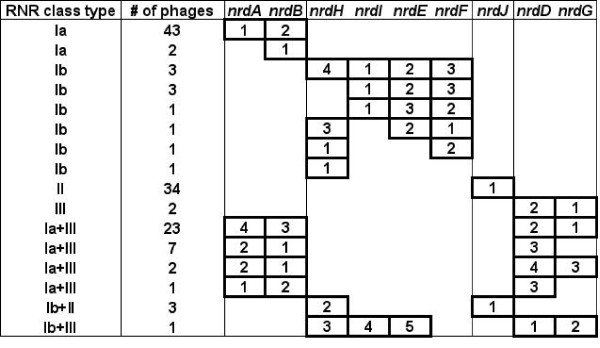
**Organization of RNR genes in 128 completely sequenced phage genomes with the number of phages containing each gene combination shown. **The number inside each cell represents the order in which the genes appear in the sequenced phage genomes.

A total of 291 RNR homologs were identified in the completely sequenced phage genomes. The majority of the phage genomes containing a given RNR class encode the entire suite of genes for that class; however, some phage genomes only contain a subset of the genes (Figure 3; Additional file 1: Table S1). To assess the conservation of gene order and gain insight into the acquisition and evolution of phage-encoded RNRs, the suite of RNR genes contained in each phage genome was examined in detail. In cellular organisms, RNR genes are typically located close together in operons, with the gene order *nrdAB* (class Ia), *nrdHIEF* (class Ib), and *nrdDG* (class III) [21]. However, many exceptions to this organization exist, as the RNR genes are sometimes separated at different locations on the chromosomes and some organisms contain split RNR genes [20,21,35,36]. In addition, some bacteria have been reported to contain two copies of class Ib RNR genes [36-38]; however, no duplicated RNR genes have been identified in phage genomes.

The organization of RNR genes identified in the 128 phage genomes is summarized in Figure 3 and described in detail for each genome in Additional file 1: Table S1. The patterns of RNR genes in phages are highly diverse, with some phages containing full suites of RNR genes organized in operons, and others lacking one or more genes belonging to a class, having genes located distantly from each other on the genome, or containing genes disrupted by introns or inteins (see below). The differences in RNR gene content and arrangement could be the result of gene gain or loss, horizontal gene transfer, recombination, or our inability to identify divergent sequences based on sequence similarity. At this time, it is unknown if the RNRs in phages containing incomplete suites of genes are functional. However, phages often contain only limited suites of genes belonging to a given host metabolic pathway, frequently representing rate-limiting steps [14,39].

Almost all of the phages with class Ia RNR genes contain both *nrdA* and *nrdB*; however, two phages only contain *nrdB* (Additional file 1: Table S1 and Figure 3). In many cases, the class Ia genes are arranged tightly together as an operon; however, these genes are sometimes interspersed with genes encoding proteins of unknown function, describing cases where *nrdA* and *nrdB* were either acquired independently or were acquired together but later separated by gene insertion.

In contrast to class Ia, the majority of the phages encoding class Ib RNR do not contain the complete suite of *nrdHIEF* genes. Interestingly, the gene order *nrdHIEF* most commonly observed in cellular organisms was never observed in phages. All phages with the complete suite of genes contained the *nrdIEFH* organization, suggesting they were probably acquired in one transfer. Accordingly, the second most common class Ib gene combination observed was a subset of this gene suite with the same order, *nrdIEF*. Phages only containing the two class III RNR genes have the gene order *nrdGD* in their genomes. In phages containing both *nrdD* and *nrdG*, these genes are typically located together in an operon; however, many phages contain only the *nrdD* gene, along with both class Ia genes (*nrdAB*). Phages containing a combination of class Ia and class III RNR show a diverse range of gene orders (Figure 3). In genomes containing the *nrdGDBA* arrangement, the *nrdGD* operon was found either directly adjacent to (e.g., *Aeromonas* phage 65), or distant from (e.g., Enterobacteria RB phages) the *nrdBA* operon. In some Enterobacteria phages (e.g., SPC35, EPS7 and T5) and *Vibrio* phage ICP1, the genomic region between the class III and class I RNR genes encodes the phosphate inducible *phoH* gene in addition to several hypothetical proteins. Similar to RNR, *phoH* is an auxiliary metabolic gene that is widespread amongst phage genomes [6]. The presence of multiple AMGs in close proximity suggests that horizontally transferred genes acquired for environmental adaptation may be localized to specific genomic islands. This arrangement has been observed previously in T4-like cyanophages where AMGs involved in photosynthesis, carbon and nucleotide metabolism are positioned closely in the genome as mobile gene cassettes [40].

### Split RNR genes in phages

**Figure 4:**
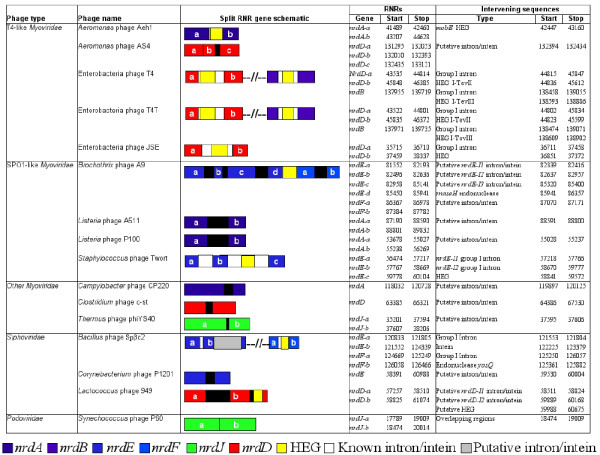
**Split RNR genes identified in completely sequenced phage genomes. **The intervening sequences (introns, inteins, and homing endonucleases (HEG)) within and between closely situated RNR genes are also listed. The RNR genes are schematically represented in the third column. The alphabetical order within each gene identifies the separated coding fragments; for example, the two regions of *nrdA *are designated as *nrdA-a *and *nrdA-b *due to their split by an intein in *Aeromonas *phage Aeh1.

Some phage genomes contain self-splicing group I introns within genes involved in nucleotide metabolism (e.g., [41-49]). Among the nucleotide metabolism genes known to contain self-splicing group I introns, RNR is the most frequently interrupted gene. In contrast to introns, very few inteins have been reported in phage RNRs [50,51]. The presence of an intron or intein is a rare evolutionary event and is unlikely to be due to chance, especially when multiple introns and inteins are discovered within the same gene. Some phage introns offer mobility because of the presence of a homing endonuclease gene (HEG) [52,53]. However, phage genomes also contain many free-standing endonuclease genes that are not encoded within introns or inteins [53-55]. Amongst the 128 phage genomes containing RNR genes, split RNR genes were observed in 16 phage genomes (Figure 4). *nrdD* was the most common split gene identified (6 cases), followed by *nrdA* and *nrdE* (4 cases each), then *nrdF, nrdB*, and *nrdJ* (2 cases each). No interrupted *nrdG, nrdH*, or *nrdI* genes were found. The high prevalence of split genes amongst the RNRs of cultured phages described here has also been observed in a bioinformatic study of uncultured phage communities that reported 7 *nrdA* and 2 *nrdJ* subunits interrupted by inteins in the Global Ocean Sampling environmental metagenome data [56].

**Figure 5:**
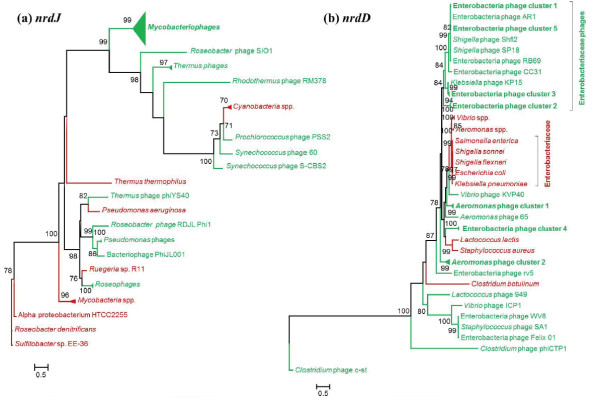
**Class II and III RNR protein phylogenies. **Phylogenetic trees constructed by the Maximum likelihood method (PhyML) based on the aligned amino acid sequences of **a)***nrdJ*: the collapsed clades include: Cyanobacteria spp. (*Prochlorococcus marinus*, *Synechococcus *sp. PCC 7335 G and WH 7803), Mycobacteriophages (Airmid, Backyardigan, Benedict, Bxz2, Che12, D29, DaVinci, Eagle, EricB, George, Gladiator, Hammer, JHC117, Microwolf, PackMan, Peaches, Pukovnik, RedRock, Rockstar, Shaka, and Vix), Mycobacteria spp. (*Mycobacterium *sp. MCS and *M. tuberculosis*), *Pseudomonas*phages (YuA and M6), Roseophages (DSS3P2 and EE36P1), and *Thermus*phages (P23-45, P74-26, and phiYS40), and **b)***nrdD*: the collapsed clades includes *Aeromonas* phage cluster 1 (Aeh1, phiAS5, and PX29), *Aeromonas*phage cluster 2 (25, 31, 44RR2.8 t, and phiAS4), *Aeromonas *spp. (*A. hydrophila* and *salmonicida*), Enterobacteria phage cluster 1 (RB51, RB14, and RB32), Enterobacteria phage cluster 2 (Phi1, RB49, and JSE), Enterobacteria phage cluster 3 (RB16 and RB43), Enterobacteria phage cluster 4 (EPS7, SPC35, and T5), Enterobacteria phage cluster 5 (T4 and T4T), and *Vibrio *spp. (*V. cholerae* and *parahaemolyticus*). The phages and bacterial hosts (both taxon labels and nodes) are color coded in green and red, respectively. The bootstrap values are shown for nodes with ≥ 70% support. The scale bar represents the number of amino acid substitutions per site.

Amongst the T4-like viruses, split RNR genes are found in the phages T4 and T4T that infect *Escherichia coli*. In these phages, the *nrdB* gene is interrupted by a self-splicing group I intron that contains a HNH homing endonuclease, named I-TevIII [47]. Free-standing homing endonucleases, *mobE*, are also commonly inserted between the well-conserved class Ia genes of T4 and related RB phages [57,58]. In some T-even phages, the *nrdD* gene is interrupted by a group I intron, I-TevII, with an active truncated homing endonuclease gene [59]. Another well-studied example is the *nrdA* gene of *Aeromonas* T-even phage Aeh1, which is encoded by two separate genes (*nrdA-a* and *nrdA-b*) due to the insertion of a *mobE* homing endonuclease [60]. Despite the absence of a self-splicing intron, this *nrdA* gene is still functional [60]. Another T4-like-phage, *Aeromonas* phage phiAS4 also contains a fragmented class III RNR gene [61]. The *nrdD* gene of phiAS4 consists of three broken coding regions, where the first two overlap by 40 nt and the last two are separated by a non-coding sequence (41 nt in length). For the latter region, no similarities to inteins were found, but a BLAST search revealed 98% nucleotide similarity to a fraction of the uninterrupted *nrdD* gene of *Aeromonas* phage 25. The *nrdD* of *Aeromonas* phages phiAS4 and 25 share 97% overall nucleotide identity with noticeable gaps near the breaking points of the *nrdD* split gene in phiAS4. Their phylogenetic grouping (Figure 5b) also suggests that the *nrdD* genes of these phages share a recent common ancestor; however, the process that led to fragmentation of the phiAS4 *nrdD* gene remains unknown.

**Figure 6:**
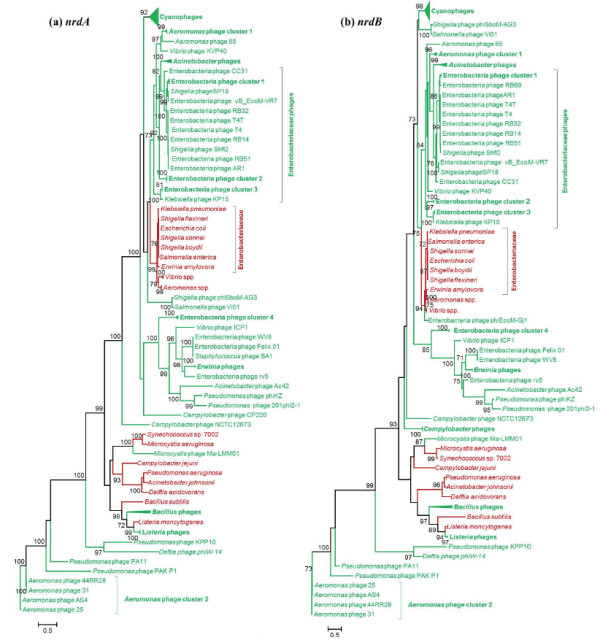
**Class Ia RNR protein phylogenies. **Phylogenetic trees constructed by the Maximum likelihood method (PhyML) based on the aligned amino acid sequences of **a)***nrdA *and **b)***nrdB*. In both trees the collapsed clades include *Aeromonas *phage cluster 1 (Aeh1, phiAS5, and PX29), *Aeromonas *phage cluster 2 (25, 31, 44RR2.8 t, and phiAS4), *Aeromonas *spp. (*A. hydrophila *and *salmonicida*), *Bacillus *phages (SPO1 and 0305phi8-36), *Campylobacter* phages (CP220 and Cpt10), Cyanophages (*Prochlorococcus* phages P-HM1, P-HM2, P-RSM4, P-SSM2, P-SSM4, P-SSM7, *Synechococcus *phages S-CRM01, S-PM2, S-RSM4, S-ShM2, S-SM1, S-SM2, S-SSM5, S-SSM7, syn1, syn9, syn19, and syn33), *Acinetobacter *phages (133, Acj61, and Acj9), Enterobacteria phage cluster 1 (JS98, JS10, and IME08), Enterobacteria phage cluster 2 (RB49, Phi1, and JSE), Enterobacteria phage cluster 3 (RB16 and RB43), Enterobacteria phage cluster 4 (EPS7, SPC35, and T5), *Erwinia *phages (phiEa 104 and phiEa-214), *Listeria *phages (P100 and A511), and *Vibrio *spp. (*V. cholerae *and *parahaemolyticus*). The phages and bacterial hosts (both taxon labels and nodes) are color coded in green and red, respectively. The bootstrap values are shown for nodes with ≥ 70% support. The scale bar represents the number of amino acid substitutions per site.

Amongst the SPO1-like *Myoviridae*, an interesting split gene example is the *nrdA* gene of *Listeria* phages A511 [62] and P100 [63], which have fragmented coding regions separated by a non-coding sequence 210 nt in length. A BLAST similarity search of the non-coding region against the intein database suggests traces of a putative intein. The complete *nrdA* sequences (including the intervening sequence) share almost 97% nucleotide identity in the two phages. Therefore, it is likely that the *nrdA* gene was interrupted by an intein in the lineage ancestral to *Listeria* phages A511 and P100, an idea that is supported by their phylogenetic placement (Figure 6). Interestingly, the protein coding regions are more similar to the intact *nrdA* of their bacterial host *Listeria monocytogenes* (~70% amino acid identity) than to other SPO1-like phages, suggesting that these *Listeria* phages may have acquired the class Ia genes from their host. Moreover, when the intergenic sequences of the *Listeria* phage *nrdA* genes were compared against the NCBI non-redundant database, nucleotide-level similarities were found to repeat regions interspersed throughout the *Brochothrix* phage A9 genome. *Brochothrix* phage A9 is a SPO1-related *Myoviridae* sharing significant similarities with *Listeria* phages and *Staphylococcus* phage Twort [64]. This evolutionary relatedness is also observed amongst their bacterial hosts; the genus *Brochothrix* is closely related to the *Listeria* genus [65]. *Brochothrix* phage A9 *nrdE* and *nrdF* genes are also interrupted by multiple intervening sequences [64]. The putative *nrdE* and *nrdF* proteins of this phage are encoded by four and two fragmented open reading frames separated by three introns, an intein-encoded homing endonuclease, and an intron, respectively (Figure 4). The sequence separating the *nrdA* coding regions in *Listeria* phages matches one of the introns present within the *nrdE* gene of *Brochothrix* phage A9, potentially indicating genetic exchange between *Brochothrix* and *Listeria* phages during a mixed infection. The related SPO1-like *Staphylococcus* phage Twort also has an interrupted *nrdE* that contains two self-splicing group I introns inserted near functionally important and highly conserved amino acid residues [45]. One of the two introns encodes a functional HNH homing endonuclease, suggesting endonuclease-mediated transposition [45]. Therefore, the presence of multiple introns in the class Ib genes of SPO1-like phages does not necessarily imply gene disruption; in fact, these introns could be beneficial to phages by regulating splicing [66].

Several other cases of split genes were observed in *Myoviridae* infecting *Campylobacter*, *Clostridium*, and *Thermus*. *Campylobacter* phage CP220 has an *nrdA* interrupted by a putative self-splicing intein [67]. The genome of *Campylobacter* phage CP220 is highly related to *Campylobacter* phage Cpt10 (>97% genome-wide nucleotide identity). Despite the overall relatedness of these genomes, no *nrdA* gene was identified in the *Campylobacter* phage Cpt10 genome through HMMER searches. Manual analysis of the genomic neighborhood of the *nrdB* gene in the Cpt10 genome revealed the highly interrupted remnant of an *nrdA* gene, disrupted by two hypothetical proteins and an intein [67]. The striking differences in the *nrdA* gene of these closely related genomes suggest distinct evolutionary selection pressures on this RNR gene in the two phages. The identical *nrdAB* positions and high *nrdB* sequence similarity (97% at the nucleotide level and 99% at the protein level) between phages CP220 and Cpt10 suggest that both the *nrdA* and *nrdB* genes were present in the ancestral lineage before they diverged. At some later point, the *nrdA* gene was interrupted in both genomes, perhaps before the speciation event (since CP220 also contains a putative intein domain). However, after these genomes diverged, the *nrdA* of phage Cpt10 was further interrupted by hypothetical proteins, resulting in the present pseudogene. Unlike the recently sequenced *Campylobacter* phage vB_CcoM-IBB_35 [68], both Cpt10 and CP220 lack class III RNR, supporting the suggestion that phage vB_CcoM-IBB_35 has a broader host range. Interestingly, the *nrdA* of phage vB_CcoM-IBB_35 is also interrupted by two putative inteins [68] with 88% and 58% nucleotide identity to the *nrdA* of Cpt10 and CP220 phages, respectively. This indicates that the *nrdA* of *Campylobacter* phages is not being maintained; instead it appears to be degenerating and propagating inteins and other proteins. It is unknown whether the *nrdA* genes of these *Campylobacter* phages are still functionally active. In addition, the *nrdD* gene of *Clostridium* phage c-st is interrupted by a putative intein [69] and shares 97% nucleotide identity to the *nrdD* of the unclassified *Clostridium* phage D-1873 contig (data not shown since this is not a completely sequenced phage genome) which is also interrupted by a putative intein domain. Therefore, it is likely that this particular gene was interrupted by a self-splicing intron or intein in the most recent lineage ancestral to c-st and D-1873. Finally, previous studies have reported a split class II RNR in *Thermus* phage phiYS40, where the *nrdJ* gene is encoded by two consecutive open reading frames (*nrdJ-a* and *nrdJ-b*) separated by a short 12 nucleotide intergenic region [20,70].

Several examples of split RNR genes were also observed amongst *Siphoviridae*. *Bacillus subtilis* phage Spβc2 has an *nrdE* gene that contains both a group I intron and an intein coding sequence, as well as an *nrdF* that contains an intron encoding a homing endonuclease, *yosQ*[51]. *Corynebacterium* phage P1201 contains an *nrdE* gene interrupted by a CP1201 RIR1 intein; however, the *nrdE* gene of this phage has been experimentally demonstrated to be functionally active [71]. In *Lactococcus* phage 949, the *nrdD* gene is interrupted by a non-coding intron, resulting in two distinct coding regions (*nrdD-a* and *nrdD-b*). The NrdD-b coding region of this phage also harbors putative HEG domain remnants of an endonuclease gene and an intein that is similar to the Chy RIR1 intein found in RNR genes of thermophilic bacteria. Additionally, phage 949 contains a group I intron between the aerobic class Ib (*nrdHIE*) and anaerobic class III (*nrdDG*) genes [72]. The two hypothetical proteins situated next to this intron also show traces of inteins such as Pho RIR1 and Chy RIR1 that are typically found within the RNR genes of cellular organisms (data not shown). Multiple homing endonucleases, particularly encoded within introns, in the genes of *Lactococcus* phages have been reported [72,73]. We hypothesize that these hypothetical proteins between RNRs are putative introns or inteins encoding yet unknown endonucleases, allowing lateral transfer of themselves and surrounding genomic regions between phages. Horizontal transfer of RNRs could present an evolutionary advantage to recipient phages under certain environmental conditions. Amongst the few *Podoviridae* containing RNRs*,* the only split gene case observed was the *nrdJ* of *Synechococcus* phage P60, where the two coding regions (*nrdJa/Jb*) overlap by 334 nucleotides [4,20].

### Phylogenetic analysis of phage and host RNR genes

**Figure 7:**
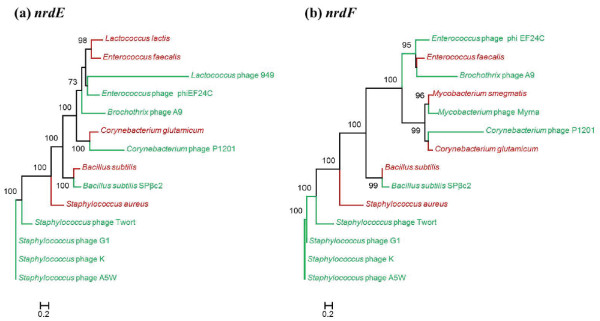
**Class Ib RNR protein phylogenies. **Phylogenetic trees constructed by the Maximum likelihood method (PhyML) based on the aligned amino acid sequences of **a)***nrdE *and **b)***nrdF*. The phages and bacterial hosts (both taxon labels and nodes) are color coded in green and red, respectively. The bootstrap values are shown for nodes with ≥ 70% support. The scale bar represents the number of amino acid substitutions per site.

Previous individual gene and genomic-signature based phylogenies have shown that phages either cluster near their bacterial hosts, supporting their co-evolution (e.g., [14,74-111]) or cluster separately from their bacterial hosts, suggesting that they are evolving independently (e.g., [5,6]). To investigate the diversity and evolutionary history of RNR genes, phylogenies were reconstructed using proteins encoded by the completely sequenced phage genomes along with sequences from their known bacterial hosts or closely-related representative bacterial species in cases where host sequences are not available (Additional file 2: Table S2). Figures 5, 6, and 7 show PhyML trees for selected class Ia (*nrdAB*), class Ib (*nrdEF*), class II (*nrdJ*) and class III (*nrdD*) proteins. Phylogenetic analyses of RNRs in phages and their known bacterial hosts present four potential evolutionary scenarios. It should be noted that these data are limited by a lack of knowledge regarding the actual host range of each phage and the fact that sequences are not available for all potential bacterial hosts. Hence, we would like to emphasize that these evolutionary scenarios are subject to change as more sequence data from phages and their associated hosts are accumulated.

1. *Phages infecting a common host cluster together, with their bacterial hosts forming a sister group*. This scenario is demonstrated by several strongly supported close relationships (100% bootstrap support). For example, class Ia genes in *Listeria* phages A511 and P100 cluster with their host *Listeria monocytogenes* and *Microcystis* phage Ma-LMM01 groups with cyanobacteria (Figure 6). Phages infecting members of the *Enterobacteriaceae* family (*Escherichia*, *Erwinia*, *Shigella*, *Salmonella*, and *Klebsiella* spp.) also group together and form a sister clade with their hosts (Figure 5b and 6a-b). Although present in fewer phages, the phylogenies of class Ib RNRs (Figure 7) also mainly display clustering of phages with the hosts they infect. For instance, *Bacillus* phage SPβc2 groups with *Bacillus subtilis*, *Corynebacterium* phage P1201 clusters with host *Corynebacterium glutamicum*, and Mycobacteriophage Myrna groups with *Mycobacterium smegmatis*. This phylogenetic grouping suggests recent horizontal gene transfers between these phages and their isolation hosts. There is also a strong possibility that phage RNRs have ameliorated to conform to host genome signatures during phage-host co-evolution, providing a selective advantage [76]. Other interesting examples include the clustering of the *nrdE* of *Lactococcus* phage 949 and *Enterococcus* phage phiEF24C, with their respective hosts *Lactococcus lactis* and *Enterococcus faecalis* forming a sister clade (Figure 7a; 60% bootstrap support) and the *nrdJ* of cyanophages *Prochlorococcus* phage PSS2 and *Synechococcus* phages P60 and S-CBS2 clustering together near a cyanobacterial host clade (Figure 5a). These examples are consistent with the co-evolution model, in which phage and host RNR genes are closely related, but sufficiently different to allow discrimination between phages and hosts.

2. *Phages infecting similar hosts cluster together independent of their bacterial hosts*. The class Ia RNR genes (Figure 6) of cyanophages infecting *Synechococcus* and *Prochlorococcus* spp. form a strongly supported clade (>90%) that is distant from the class Ia genes encoded by cyanobacteria. Known *Prochlorococcus* spp. and the majority of the *Synechococcus* spp. in the RNRdb only contain class II *nrdJ*. This suggests that either these cyanophages either acquired class Ia RNR genes from a different host than the one on which they were initially cultured, or that these genes were acquired through lateral gene transfer with other phages. Class II *nrdJ* genes (Figure 5a) of mycobacteriophages form a distinct monophyletic clade (99% support) separate from *Mycobacteria* spp. Likewise, anaerobic class III RNRs (Figure 5b) of phages infecting *Vibrio, Lactococcus, and Staphylococcus* are only distantly related to their hosts. This evolutionary pattern implies that phages are evolving through a combination of vertical inheritance and lateral gene transfer with other phages, and indicates that phage RNRs are under different selection pressures than host RNRs.

3. *Phages infecting similar hosts form several separate clusters that are distant from their hosts*. This scenario is demonstrated by class Ia, class II, and class III RNR genes of phages infecting *Aeromonas*, *Acinetobacter, Pseudomonas*, *Vibrio*, and *Thermus* species, as well as Enterobacteria*.* These phages do not form a monophyletic clade or group with their known bacterial hosts. Instead, these phages belong to separate clusters that are scattered across different parts of the trees (Figure 5 and 6). For example, *Aeromonas* phages form two separate clusters (cluster 1 & 2), with an additional phage (*Aeromonas* phage 65) falling outside of the two clusters. *Aeromonas* phages contain both aerobic class Ia (*nrdA* and *nrdB*) and anaerobic class III (*nrdD* and/or *nrdG*) genes. The *nrdD* tree (Figure 5b) is congruent with the *nrdAB* trees (Figure 6) with respect to the clustering of *Aeromonas* phages. *Aeromonas* phage clusters 1 and 2 consist of phages with the *nrdBAD* and *nrdGDBA* gene arrangement, respectively. The different patterns of RNR organization in *Aermomans* phages suggest that *nrdAB* and *nrdDG* were acquired in a single event from a common ancestor and that subsequent horizontal gene transfer and recombination events disrupted this arrangement, or that *nrdAB* and *nrdDG* were acquired independently in multiple instances by horizontal transfer. Likewise, several independent well-supported groups of phages infecting Enterobacteria were observed in Figures 5b and 6. The Enterobacteria phages belonging to clusters 1, 2, and 3 contain the same RNR gene organization (*nrdGDBA*), while clusters 4 and 5 contain different gene orders. These patterns suggest that each phage cluster is evolving differently and perhaps with limited horizontal transmission between phage clusters and Enterobacterial hosts. Closely-related *Thermus* phages P23-35 and P74-26 group together with 97% bootstrap support, but are phylogenetically closer to T4-like marine phages than to other *Thermus* phages or their host. The two *Vibrio* phages containing class I+III genes with different arrangements do not cluster together; KVP40 is nested with the *Enterobacteriaceae* host clade while ICP1 groups with related Enterobacteria phages. These examples strongly support the hypothesis that RNRs were acquired by each group independently from their hosts in one or more events followed by horizontal gene transfer and subsequent changes within each cluster of related phages.

4. *Phages infecting the same host cluster together and are more related to different bacteria rather than their known hosts*. For example, the *nrdD* of Enterobacteria phage cluster 4 (EPS7, SPC35, and T5) groups with *Lactococcus* and *Staphylococcus* bacterial species (Figure 5b) and the *nrdF* of *Brochothrix* phage A9 infecting *Brochothrix thermosphacta* clusters with *Enterococcus faecalis* (Figure 7b). However, the bootstrap support values are weak (50%), suggesting that these groupings are unreliable and could change as sequences from closely related phages and representative hosts become available. Additionally, the *nrdJ* of *Thermus* phage phiYS40 groups with *Pseudomonas aeruginosa* with good bootstrap support (82%) (Figure 5a). Phage phiYS40 proteins share similarity to proteins from diverse dsDNA phages and bacteria and have been suggested to result from multiple recombination events between phages and their hosts [70]. It is possible that this phage has a broad host range and underwent gene exchange with *Pseudomonas* spp. as well. Along these lines, it is also important to note that the actual extent of the host range for each of these cultured phages is unknown, making it possible that the phages also infect hosts other than the one on which they were originally cultured.

Since RNRs often exist in operons, we assessed the co-inheritance of different RNR proteins in phages. In phages containing both class Ia genes, *nrdA* phylogeny (Figure 6a) is topologically congruent with *nrdB* phylogeny (Figure 6b). Combined with the fact that these genes frequently occur together in phages (Figure 3), where they often co-localize to the same operon (Additional file 1: Table S1), this finding suggests that *nrdA* and *nrdB* were acquired by phages simultaneously. In addition, phylogenetic trees reconstructed using class Ib (Figure 7a-b) and class III (Figure 5b) RNRs show congruency, and phages with class combinations I+II and I+III have congruent clustering patterns. Together, these results suggest that genes belonging to different RNR classes are often acquired simultaneously and share the same evolutionary history.

### RNRs in viral metagenomes

**Figure 8:**
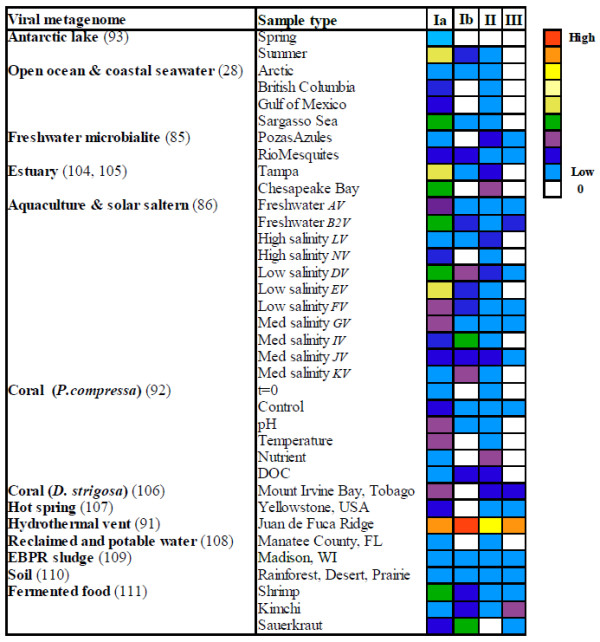
**RNRs in viral metagenomes. **Heatmap representation of the relative abundance of RNR classes among sequenced viral metagenomic data sets, ranging from low abundance (blue) to high abundance (red). The normalized abundance values are summed over all genes belonging to each RNR class. Viral metagenomes with ≤ five RNR hits are not shown [28,86,91-93,104-111].

RNRs are among the most frequently identified genes in natural communities [28,29,77]; therefore, this study determined the abundance and distribution of each RNR class in all available environmental viral metagenomes (reviewed in [81]). Since viral metagenomes are not affected by culturing biases, analyzing metagenomic sequences enables a more accurate determination of the types of RNR present in natural phage communities, as well as an examination of how RNR distribution changes under varying environmental conditions. Investigating RNR genes in viral metagenomes presents a major challenge because of the relatively short read lengths, inability to definitively link specific metagenomic reads to the same phage genome, and the high variability in DNA extraction, sequence amplification, metagenome preparation, and sequencing methods [78-80]. To overcome some of these limitations, the FragGeneScan program with model parameters based on the sequencing read type and error rate was used to predict the protein-coding regions from 22 environmental viral metagenomes [81]. Hidden Markov Models (HMM) were then used to identify similarities to RNR gene profiles and the data was normalized by metagenome size and gene length. The relative abundance of each RNR class in each viral metagenome is shown as a heatmap in Figure 8. Of all the viral metagenomes analyzed, only two (rice paddy soil from Korea [82] and hydrothermal vents from the East Pacific Rise [75]) did not contain any identifiable RNR homologs. Several viral metagenomes (coastal sediment and seawater from California [83,84], marine microbialites from Highborne Cay [85], a solar saltern pond with medium salinity (MV) [86], salterns from Alicante Spain [87,88], activated sludge [89], and a hypersaline lake [90]) contained ≤ five RNR hits and were therefore excluded from the analysis to ensure robustness of results.

The RNR class distribution amongst phages in different environments is likely related to the types of host bacteria found in that ecosystem. In particular, the relative abundance of aerobic versus anaerobic RNR classes would be expected to correspond with the oxygen requirements of the dominant hosts present. In most environments, class Ia RNR sequences were the most abundant, followed by class II, class Ib, and then class III. All viral metagenomes with RNR genes contain class Ia genes, and the vast majority also contain class II genes. Class Ib and class III RNRs were detected in significantly fewer metagenomes, although it should be noted that the environments from which the metagenomes were sequenced are highly biased and dominated by aerobic, aquatic samples. Class III RNRs are absent in most marine metagenomes, but appear more frequently in freshwater metagenomes (including freshwater microbialites and aquaculture ponds), hot springs, fermented foods, and low/medium salinity solar salterns (Figure 8).

Interestingly, the viral metagenome containing the highest normalized abundance of RNR genes (in all classes) originated from a hydrothermal vent from Juan de Fuca Ridge [91]. The majority (>75%) of the identifiable phage sequences in this vent metagenome are similar to *Myoviridae*[91]. The high number of RNR genes detected in this viral metagenome may be due to the fact that *Myoviridae* frequently contain RNR genes (see above), combined with the fact that the metagenome reads in this study are fairly long (average = 334 bp) compared to the other metagenomes. However, the only other available hydrothermal vent metagenome (from the East Pacific Rise; [75] does not contain any RNR genes, suggesting that high RNR abundance may not be a general feature of hydrothermal vents and may instead reflect the biogeochemistry and resulting effects on phage and host types in individual vent systems. In contrast to the *Myoviridae*-dominated Juan de Fuca Ridge metagenome, the East Pacific Rise vent was dominated by temperate viruses, potentially explaining the difference in RNR abundance. However, it is important to note that the East Pacific Rise metagenome contained an extremely small number of sequences; therefore, future research is needed to determine the prevalence of RNR in phages from hydrothermal vents.

The heatmap representation (Figure 8) also demonstrates changes in the distribution of RNR classes in multiple metagenomes from the same habitat that differ in terms of the geographical location (e.g., marine samples from the Arctic versus the Gulf of Mexico), sampling season (e.g., Antarctic freshwater lake in spring versus summer), or physicochemical properties (e.g., corals exposed to different pH, temperature, or carbon and nutrient concentrations). Regardless of location, all seawater metagenomes [28] contain class Ia and class II RNR genes and lack class III genes. However, the different marine locations demonstrate differences in the relative abundance of RNR classes, and only the Arctic and the Sargasso Sea contain class Ib genes. Viral metagenomes associated with the coral *Porites compressa* under various experimental treatments (controls, increased temperature, decreased pH, increased nutrients, and increased dissolved organic carbon (DOC); [92] show differences in the relative abundance of RNR classes resulting from shifts in the composition of the host and phage communities in response to the treatments. The viral metagenomes from the freshwater Antarctic lake Limnopolar in two different seasons (spring and summer; [93] are quite distinct. RNRs are very rare in the spring metagenome, a finding that can be explained by the large proportion of single-stranded DNA viruses infecting eukaryotes in the lake during this time [93]. The presence and higher abundance of class I and II RNR genes in the same lake during the summer reflect a shift in the viral community towards dsDNA *Caudovirales* infecting bacteria [93]. Amongst the viral metagenomes sequenced from freshwater aquaculture ponds and solar salterns of differing salinity [86], a wide variety of RNR class patterns were observed. Within a given aquatic environment at different time points, differences in the RNR composition of the viral community were observed (e.g., freshwater aquaculture pond AV and B2V; low salinity EV and DV or FV). The available examples indicate that the type of RNRs favoring phage success change over time in response to changing environmental conditions, presumably as a reflection of the availability and activity of particular bacterial hosts. Data from the viral metagenomes indicate that RNRs in uncultured viral communities are highly diverse and dynamic, and that further work is needed to understand the role of phage-encoded RNRs in the environment and the conditions under which they provide a selective advantage.

While surveying viral metagenomes for RNR homologs provides some insight into the RNR content of uncultured phage communities, there are many limitations to this method. The typical lengths of metagenomic sequence reads generated using 454 pyrosequencing are ~100-400 bp, and those generated with Sanger sequencing are ~500-1000 bp. Compared with the length of RNR genes (ranging from 200–3000 bp), it is likely that many of the RNR genes in these fragmented reads will be missed, partially predicted, or non-distinguishable because of shared sequence domains. The short length of metagenomic reads also means that highly divergent proteins are unlikely to be recognized. Alternatively, a short read containing a motif that is highly conserved between an RNR gene and a gene of different function (e.g., thioredoxin/glutaredoxins, pyruvate formate-lysate enzymes) may be misidentified, leading to false positive results. Additionally, since it is impossible to determine which metagenomic reads originate from the same phage genome, the presence of RNR class combinations (as seen in the cultured phage genomes) cannot be predicted for natural viral communities. Other sources of inaccuracy in the metagenome analysis could result from differences in metagenome sizes or read length, as well as the presence of contaminating non-viral sequences (e.g., from bacterial hosts or free DNA). Sequencing of more viral metagenomes in the future will enable a better understanding of RNR distributions in uncultured phage communities, allowing insight into the environmental conditions under which encoding RNRs provides a selective advantage to phages.

## Conclusions

RNRs play a central role in metabolism in all cellular domains of life, yet these enzymes have not yet been examined systematically in phages. Here we presented a bioinformatic analysis of RNRs amongst 685 completely sequenced dsDNA phage genomes and 22 environmental viral metagenomes. Approximately nineteen percent of the phage genomes contained one or more RNR genes, with RNR distribution patterns corresponding to phage type, original source of phage isolation, and the known host’s oxygen utilization ability. In addition, analysis of viral metagenomic data revealed diverse and dynamic RNRs in uncultured environmental viral communities. Phylogenetic analyses suggested multiple possible scenarios for the acquisition and evolution of RNRs in phages and supported a prominent role for horizontal gene transfer. In conclusion, this comprehensive analysis provided insight into the diversity, distribution, and evolutionary history of phage-encoded RNRs, paving the way for future studies to investigate the role of RNRs in phage genomes and the environmental conditions under which these auxiliary metabolic genes provide a selective advantage to phages.

## Methods

### Data collection

The protein coding sequences of 685 completely sequenced dsDNA phage genomes were downloaded from the PhAnToMe phage database (http://www.phantome.org/) in July 2011. The phage metadata (phage family, original environment of isolation, bacterial host, and oxygen requirements of known hosts) were compiled from primary literature and public databases (e.g., NCBI, Mycobacteriophage database, and the Felix d’Herelle Reference Center for Bacterial Viruses). The DNA viral metagenomes from 22 environmental samples were obtained from their respective databases as listed in Rosario and Breitbart (2011) and each metagenome was de-replicated to remove duplicate sequences. The protein-coding regions were predicted from the short sequence reads of the viral metagenomes using FragGeneScan v1.16 [94] with model parameters selected based on the sequencing read type and lowest error rate.

### RNR identification in phage genomes and viral metagenomes

Protein homologs of all three RNR classes: class Ia (encoded by *nrdA* and *nrdB*), class Ib (encoded by *nrdE, nrdF, nrdH,* and *nrdI*), class II (encoded by *nrdJ*), and class III (encoded by *nrdD* and *nrdG*) were identified in proteins predicted from the phage genomes and viral metagenomes using HMMER v3.0 [95] with the default settings and hidden markov model (HMM) profiles developed by Lundin et al. [20]. The identified RNR protein homologs were then filtered based on an E-value cut-off of 0.0001 and manually curated to include any known RNR homologs that were not identified by HMMER (n=2, *nrdE* of *Staphylococcus* phages K and Twort, as reported in the RNRdb) and to exclude false positives resulting from similarities to other conserved domain motifs/proteins. To reduce false positives, hits to *nrdH* and *nrdG* were only accepted if they were situated close to other RNR genes on the genome, as these genes also have similarities to thioredoxins/glutaredoxins and pyruvate formate-lysate genes, respectively. If a particular sequence had hits to multiple RNR genes, the result with the lowest E-value was selected. RNR genes identified in the phage genomes were also manually screened to detect the presence of intervening sequence features based on comparisons to known RNR sequences, searches against the NCBI Conserved Domain Database (http://www.ncbi.nlm.nih.gov/Structure/cdd/cdd.shtml; [96], and BLAST comparisons against the New England Biolabs Intein database (InBase: http://tools.neb.com/inbase/; [97]. Finally, the number of homologs to each RNR gene in the viral metagenomes was normalized by the total metagenome size and the length of the RNR gene (in amino acid residues). Normalized abundance data from the metagenomes was then plotted against the RNR classes and displayed as a heatmap.

### Phylogenetic analysis

Phylogenetic analyses were performed separately for each RNR gene. Phylogenies of the reductant *nrdH* (class Ib), flavoprotein *nrdI* (class Ib), and activase *nrdG* (class III) are not shown since these proteins are too short to be informative, present in a very limited number of phages, and are not catalytic or radical-generating subunits. Amino acid sequences of phage RNRs were aligned with corresponding sequences from their known bacterial hosts or related representative bacteria (Additional files 1 and 2) with the PRALINE profile based alignment tool [98]. For alignments with a large number of gaps, conserved blocks were selected from the multiple protein sequence alignments using GBLOCKS [99,100]. Phylogenetic analysis was performed with the maximum likelihood method using PhyML v3.0 [101]. The best amino acid substitution model for each RNR protein alignment was selected with the Akaike Information Criterion (AIC) approach using ProtTest v3.0 [102]. The number of aligned positions and model parameters used to build the RNR phylogenetic trees are detailed in Additional file 3: Table S3. Phylogenetic trees were built starting with the BioNJ tree and optimized with the Nearest Neighbor Interchange (NNI) search algorithm in PhyML with default options. Bootstrap support values of the PhyML protein trees were computed by re-sampling 1000 times. Phylogenetic trees were edited and visualized using MEGA v5 [103].

## Competing interests

The authors declare that they have no competing interests.

## Authors’ contributions

MB and RAE conceived the study. BD and MB designed the study. BD and BX collected, compiled and analyzed the data. BD performed the research, interpreted the results, and wrote the manuscript. DL provided the RNR HMM profiles and helped with the identification of RNR protein homologs in phages. MB coordinated the work and helped write the manuscript. All authors read, edited, and approved the final submitted manuscript.

## Supplementary Material

Additional file 1: Table S1 List of phages containing RNRs and summary of phage related factors used in this study. Gene order (5’-3’) and strand information is based on the direction the genome was deposited in GenBank. Click here for file

Additional file 2: Table S2 List of representative bacterial hosts and RNR protein GI numbers used in the phylogenetic tree construction. Click here for file

Additional file 3: Table S3 List of total number of sequences and aligned positions with best-fitted amino acid substitution model used for constructing each of the RNR protein trees. Click here for file
